# Human Exposure to Trace Elements (Al, B, Ba, Cd, Cr, Li, Ni, Pb, Sr, V) from Consumption of Dried Fruits Acquired in Spain

**DOI:** 10.3390/foods13172660

**Published:** 2024-08-23

**Authors:** Juan Ramón Jáudenes-Marrero, Soraya Paz-Montelongo, Javier Darias-Rosales, Dailos González-Weller, Ángel J. Gutiérrez, Arturo Hardisson, Carmen Rubio, Samuel Alejandro-Vega

**Affiliations:** 1 Area of Toxicology, Universidad de La Laguna, 38071 La Laguna, Spain; jjaudene@ull.edu.es (J.R.J.-M.); xavijavi96@gmail.com (J.D.-R.); dgonzal@ull.edu.es (D.G.-W.); ajguti@ull.edu.es (Á.J.G.); atorre@ull.edu.es (A.H.); crubio@ull.edu.es (C.R.); salejand@ull.edu.es (S.A.-V.); 2 Grupo Interuniversitario de Toxicología Ambiental y Seguridad de los Alimentos y Medicamentos, Universidad de La Laguna, 38071 La Laguna, Spain; 3 Laboratorio Central, Servicio Público Canario de Salud, 38006 Santa Cruz de Tenerife, Spain

**Keywords:** dried fruits, trace elements, toxic metals, risk assessment, Spain

## Abstract

Dried fruits are one of the most frequently consumed products by the population. Drying fruits prolongs their shelf life and also concentrates more nutrients. However, these products may contain dangerous levels of trace elements that can be harmful to health. The content of trace elements (Al, B, Ba, Cd, Cr, Li, Ni, Pb, Sr, V) in 42 samples of different dried fruits (dates, prunes, sultanas, dried apricot kernels, and dried figs) was determined by inductively coupled plasma spectrometry (ICP-OES). The concentrations of Al found in prunes (12.7 ± 5.13 mg Al/kg) and the concentrations of B found in dried plums (6.26 ± 4.45 mg B/kg) were significantly higher (*p* < 0.05). Regarding the risk assessment, the percentages of contribution to the maximum recommended intakes by Li in all the dried fruits studied stand out, reaching 35.3% in the case of dried plums. This study concludes that the recommended daily intake of dried fruit (30 g/day) does not pose a toxicological risk about these trace elements.

## 1. Introduction

Preserving and storing fresh fruits is generally challenging, while drying is a technique that facilitates preservation by delaying bacterial degradation [1,2,3]. Traditional preservation methods have included sun exposure, use of hot air, exposure to sulphur dioxide and vacuum packing among others [4,5].

Dried fruits are products traditionally included in the Mediterranean diet which are highly valued for their sweetness and stability [6]. Their nutritional composition is similar to that of the original products, standing out for their fibre and potassium content [7,8]. Likewise, the drying process favours the concentration of micronutrients up to 3–5 times more in dried fruits than in fresh fruits [9,10].

Among the most widely consumed dried fruits are sultanas, which are extensively used in bakery and confectionery around the world, being utilized six times more than other dried fruits [8]. Sultanas are a source of sugars, fibre, vitamins B6 and B1, and essential minerals. They have a calorie level four times higher than fresh grapes, though their vitamin C content is lower [11].

Dates, which have high nutritional value and confer health benefits such as antioxidant, anticholesterolemic, and antiatherogenic effects, are also widely used [12,13].

Prunes contain significant amounts of sorbitol, hydroxycinnamic acids, chlorogenic acids, vitamin K, and phenolic compounds [11]. Dried apricots are a source of fibre and beta-carotene and contain organic acids such as malic and citric acid and flavonoids in small amounts [11]. Finally, dried figs are rich in fibre and sugars and, although they are not rich in minerals, they contain all the essential amino acids [14].

The consumption of dried fruits can help prevent deficiency diseases, reduce chronic diseases, and maintain optimal health. The consumption of dried fruit has been correlated with a healthier diet [8,12,13,15,16]. However, due to increased environmental pollution, fruits can accumulate high concentrations of trace elements that can damage health [17]. If we consider the concentration effect that occurs when fruits are subjected to drying, the levels of these non-essential trace elements may be higher than in the original products and therefore may pose a risk to the consumers [4,18,19].

Trace elements that could be accumulated in foods and cause toxicological effects are mainly Al, B, Ba, Cd, Cr, Li, Ni, Pb, Sr, V, and Zn. Thus, these are the main elements studied in food samples for risk assessment.

Cadmium (Cd) is naturally present in the environment, but human activities have increased its background levels in soil, water, and living organisms. Among the different sources of Cd exposure, food is the main source for the non-smoking population [20]. Cadmium mainly harms the kidneys; however, it can also weaken bones and has been linked to an increased risk of cancer in several organs, including the lungs, uterus, bladder, and breasts. In the kidney, it especially affects the cells of the proximal tubules, where it accumulates over time and causes renal dysfunction [20].

Early signs of lead (Pb) poisoning, particularly from high occupational exposure or through large lead intake, often include abdominal cramps (colic), constipation, nausea, vomiting, and loss of appetite. However, the bigger threat is chronic lead poisoning as lead remains in the body for a long time [21]. Studies conducted in animal models support the plausibility of an effect of lead on blood pressure in humans. Functional renal deficits that have been associated with lead exposure include low- and high-molecular-weight proteinuria and impaired transport of organic anions and glucose. The nervous system is the main target organ for lead toxicity, with the characteristics of developmental lead toxicity being encephalopathy, decreased nerve conduction velocity, and cognitive deficits. Lead has also effects on reproduction, the immune system, and other organs [21].

Several compounds containing aluminium (Al) have the potential to produce neurotoxicity and to affect the male reproductive system. In addition, after maternal exposure, they have shown embryotoxicity and genotoxicity. Cross-linking of DNA with chromosomal proteins, interaction with microtubule assembly and mitotic spindle functioning, induction of oxidative damage, and damage to lysosomal membranes with liberation of DNAase have been suggested to explain the induction of structural chromosomal aberrations, sister chromatid exchanges, chromosome loss, and formation of oxidized bases in experimental systems [22].

Studies on humans have examined the overall harmful effects of total chromium (Cr), including death rates in general and from specific diseases like cancer (e.g., gastric tumours) [23]. It is suggested that primary exposure to Cr(VI) could cause haemotoxicity due to its accumulation in erythrocytes and subsequent reduction to Cr(III) and their binding to haemoglobin and other ligands [23].

It is evident that oxidative stress and an elevation of reactive oxygen species (ROS) are involved in the toxicity of nickel (Ni). The contribution of oxidative stress is evident in relation to reproductive toxicity, genotoxicity, immunotoxicity, and neurotoxicity [24]. It has also been postulated that nickel might exert some of its effects via perturbation of iron homeostasis [24].

Strontium (Sr) is chemically similar to calcium. Thus, the toxicity of high Sr concentrations is related to its interference in biological processes that normally involve calcium, notably skeletal development in children and neurotoxic and neuromuscular perturbations [25]. Furthermore, many calcium-dependent enzymes will function when strontium is substituted, but with changes in kinetic parameters [25].

While lithium (Li) is a well-established treatment for bipolar disorder, most research on its potential harms comes from monitoring patients on medication. Lithium has been linked to kidney problems, with nephrogenic diabetes insipidus being the most frequent side effect. Additionally, some patients on lithium experience thyroid issues, mainly in the form of asymptomatic hypothyroidism [26].

Boron (B), a semimetal, is not an essential nutrient for humans and any specific biochemical functions have not been identified. There is, however, some evidence that, in humans, B may influence the metabolism and utilization of other nutrients, especially calcium, and may have a beneficial effect on bone calcification and maintenance [26]. Excessive boron intake can lead to intoxication, causing problems in the digestive system, damage to specific kidney cells, and skin peeling. Other reported effects include seizures, circulatory failure, fluid buildup in the brain, hair loss, fatigue, loss of appetite, and mental disorientation [27].

Human and animal high exposure to soluble Barium (Ba) compounds results in a number of effects including electrocardiogram abnormalities, ventricular tachycardia, hypertension and/or and hypotension, muscle weakness and paralysis [26]. However, kidney effects, including marked severity of nephropathy, are considered the most sensitive health effect associated with long-term ingestion of Ba [26].

Vanadium (V) has not been shown to be essential for humans or possess any nutritional value. It can accumulate in the liver, kidneys, bones, lungs, and spleen [26]. Vanadium compounds may initiate some gastrointestinal problems such as diarrhoea, vomiting, general dehydration with weight reduction, intestinal inflammation, and a characteristic green tongue. High concentrations of V may cause irreversible damage to the kidneys [26].

The objectives of the present study are to determine the content of trace elements (Al, B, Ba, Cd, Cr, Li, Ni, Pb, Sr, V, and Zn) in different dried fruits (dates, prunes, sultanas, dried grapes, apricot kernels, and dried figs), to compare the concentrations found among them and with other studies, and to perform assessment of the risk derived from their consumption.

## 2. Material and Methods

Our laboratory belongs to the interuniversity group of Environmental Toxicology and Food and Drug Safety, in the Toxicology area of the University of La Laguna (Tenerife, Canary Islands, Spain). Thus, one of the main research lines of our group is the evaluation of risk and food safety, particularly in the determination of the metal profile and fluoride levels. We focus on the evaluation of foods that could be of interest to the Canarian and the Spanish populations. Since the Canary Islands are a region of Spain, the products marketed tend to be the same.

### 2.1. Samples

A total of 42 dried fruit samples were purchased from large supermarkets on the island of Tenerife (Canary Islands, Spain). Whenever possible, the samples selected were of national or European origin. In addition, all the samples selected for this study come from presentations marketed nationally, so even though the samples were acquired in an outermost region of Spain, they are representative samples of the country. Table 1 shows a breakdown of each type of dried fruit selected for this study and their origin (plums, dates, sultanas, dried apricots, and figs).

### 2.2. Samples Treatment

First, 10 g of each sample, previously homogenized, was weighed in porcelain capsules (Staalich, Werheim, Germany) and placed in an oven (Prebatem-TFT, J.P. Selecta, Barcelona, Spain) at a temperature of 70 °C for 24 h. For each sample, 3 aliquots were taken and analysed. Homogeneity tests were conducted on subsamples of the homogenized sample, revealing no significant differences between them. Subsequently, the samples were subjected to acid digestion with 65% nitric acid (HNO_3_) (Sigma Aldrich, Steinheim, Germany) and then incinerated in a muffle furnace (L40/11/B180, Nabertherm, Lilienthal, Germany) with a temperature–time programme of 450 °C for 24 h, with a progressive temperature increase of 50 °C per hour. At the end of the process, white ash was obtained, filtered, and diluted in 1.5% HNO_3_ (Sigma Aldrich, Steinheim, Germany) in a volume of 25 mL [28,29,30]. Samples were preserved in polypropylene containers and kept cold until analysis.

### 2.3. Quality Control and Analysis

The elements were determined using a Thermo Scientific iCAP PRO inductively coupled plasma optical emission spectroscopy (ICP-OES) instrument (Waltham, MA, USA). The wavelength at which each element was measured, as well as the limits of detection (LOD) and limits of quantification (LOQ) of the ICP-OES, is shown in Table 2. These were calculated from the instrumental response under reproducible conditions as ten and three times, respectively, the standard deviation of the method analysis.

The instrumental conditions of the ICP-OES were RF power of approximately 1.2 kW; gas flow (nebuliser flow, auxiliary flow) of 0.5 L/min; injection pump speed of 50 rpm; stabilisation time of 0 s. The gas used was liquid argon (99.999%, Air Liquid, Madrid, Spain).

The quality control of the method was performed with certified reference materials (SRM 1515 Apple Leaves, SRM 1548 a Typical Diet) from the National Institute of Standards and Technology (NIST). For each certified reference material, 3 aliquots were taken and analysed. In all cases, recovery percentages between 95 and 102% were obtained. In the case of Li, the standard additions method was used.

### 2.4. Statistical Analysis

The results were analysed with the Shapiro–Wilk test and the homogeneity of variance was analysed with the Levene test [31,32]. An Anova analysis was performed for data following a normal distribution, followed by the Tukey test. Non-parametric tests were performed for data that did not follow a normal distribution using the Kruskal–Wallis test and the Mann–Whitney U test [30].

The data sets statistically analysed were the concentrations of each of the elements as a function of each product, e.g., (Al (prunes), Al (dates), Al (sultanas), Al (apricot kernels), Al (figs)).

### 2.5. Dietary Intake Assessment

The estimation of dietary intake is based on the calculation of the EDI (estimated daily intake) and the percentage contribution (%), calculated by applying the following general expressions Equations (1) and (2).

(1) $$EDI\;\left(\frac{mg}{day}\right)=Daily\;consumption\left(\frac{kg}{day}\right)\cdot Concentration\;(\frac{mg}{kg})$$

(2) $$\%\;Contribution=\frac{EDI\;(\frac{mg}{day})}{Reference\;value\;(\frac{mg}{day})}\cdot 100$$

The maximum intakes of each element have been established by the main health bodies, defined, as appropriate, in terms of tolerable weekly intake (TWI), benchmark dose level (BMDL), tolerable daily intake (TDI), tolerable upper intake level (UL), oral reference dose (RfD), and provisional subchronic and chronic reference dose (p-RfD). These maximum intake limits established by EFSA (European Food Safety Authority) and WHO (World Health Organization) are set out in Table 3.

## 3. Results and Discussion

### 3.1. Trace Element Levels in the Dried Fruits

Table 4 shows the results of the determination of the trace elements under study (Al, B, Ba, Cd, Cr, Li, Ni, Pb, Sr, and V) in each of the selected types of dried fruits (dried plums, dates, sultanas, apricot kernels, and dried figs). Only these elements have been selected for determination because these are the elements with highest toxicological potential, discarding others that are considered macroelements or elements such as copper or zinc that are essential trace elements. The mean concentrations (mg/kg fresh weight), standard deviations (SD) and maximum and minimum values of the trace element determinations are specified.

The concentration of Al and B in all samples stands out. No significant differences were found between the different fruits for Li, Pb, Sr, or V, so their concentration is not highlighted in any of the samples. On the other hand, significant differences (*p* < 0.05) were found for Al, B, Ba, Cr, and Ni.

Sultanas showed significantly (*p* < 0.05) higher levels of Al than the rest of the fruits studied, with a mean concentration of 12.7 ± 5.13 mg Al/kg. Dried plums showed significantly (*p* < 0.05) higher levels of B (6.26 ± 4.45 mg B/kg), while dates showed significantly (*p* < 0.05) lower mean values (2.71 ± 0.77 mg B/kg).

Regarding Ba, figs and dates presented significant differences (*p* < 0.05) from the rest of the fruits studied, with the highest levels found in dried figs (2.32 ± 0.90 mg Ba/kg) and the lowest in dates (0.50 ± 0.32 mg Ba/kg).

Dried figs had the significantly (*p* < 0.05) highest Cd content, with mean values of 0.02 ± 0.01 mg/kg. The mean Cr content was significantly (*p* < 0.05) lower in dates, with mean levels of 0.04 ± 0.02 mg Cr/kg. Ni levels were significantly (*p* < 0.05) higher in dried figs, with levels of 0.75 ± 0.41 mg Ni/kg.

Comparing the results with those obtained in other studies, it could be highlighted that the concentrations of the elements found are generally lower, especially the concentrations of Pb and Cd [4,18,35,36,37,38]. In the present paper, only the aluminium content could be highlighted compared to the other studies presented. A comparison of the concentrations found in the different studies is shown in Table 5. It is important to add that all previous studies have been carried out in Eastern European and Eastern countries (India, Iran, Jordan, Pakistan, Serbia, and Turkey).

These differences could be due to the origin of the cultivation of these fruits, as elements such as Cd and Pb have their origin mainly in industrial environments and elements such as Al have their origin in mining environments. It should also be taken into account that food legislation in these countries may be less demanding.

### 3.2. Dietary Intake Assessment

Estimated daily or weekly intake values were calculated assuming the recommended intake of a consumption of 30 g of dried fruits per day or 210 g/week [41,42,43,44,45,46]. As the official parameters of maximum intake are weight-based, the average weight of 68.48 kg [47] was taken as a reference. Table 6 shows the results of the risk assessment.

From the consumption of 30 g/day of each of the samples analysed (plums, dates, sultanas, dried apricots, and figs), the EDI of Li of 48.3 µg Li/day (plums), 25.5 µg Li/day (dates), 43.5 µg Li/day (sultanas), 30.3 µg Li/day (dried apricots), and 37.8 µg Li/day (figs) should be highlighted. These daily intake values represent a contribution of 35.3% (plums), 31.8% (sultanas), 27.6% (figs), 22.1% (dried apricots), and 18.6% (dates) to the provisional oral reference dose (p-rfD) established by EFSA for Li [26]. This element has been identified as having an adverse renal effect, and with high chronic consumption, some additional adverse effects on thyroid function could be observed.

Al would be the second trace element with the highest percentage contribution to the maximum intake. Assuming an intake of 210 g/week, there would be an estimated weekly intake (EWI) of 2.09 mg/week (3.05%) for plums, 1.52 mg/week (2.22%) for dates, 2.67 mg/week (3.89%) for sultanas, 1.29 mg/week (1.88%) for dried apricots, and 1.73 mg/week (2.52%) for figs.

In the case of Pb, the third element with the highest contribution percentages, assuming daily rations of 30 g, there would be an EDI of 1.20 µg/day for plums, 0.60 µg/day for dates, and in the case of sultanas, dried apricots, and figs, 0.90 µg/day. In the case of lead, EFSA identifies three BMDLs, highlighting nephrotoxic and cardiovascular effects [21]. For the nephrotoxic effects, there are contribution rates of 2.78% (plums), 1.39% (dates), and 2.09% (sultanas, dried apricots, and figs), while for the cardiovascular effects, the contribution rates are 1.17% (plums), 0.58% (dates), and 0.88% (sultanas, dried apricots, and figs).

For Ni, all samples show low contribution percentages, apart from dried apricots and figs, which have estimated daily intakes of 11.7 (1.31%) and 22.7 mg/day (2.53%), respectively. The same occurs in the case of Cd, since the contribution is negligible in all cases, except for in figs, in which case an EDI of 4.20 µg/week would be made, which means a contribution of 2.45% to the TWI.

For the rest of the elements studied (Ba, Cr, Sr, B, and V), the estimated daily or weekly intakes are below 1% in all cases, which means a low contribution of these elements and a negligible risk of toxicity.

Thus, we can conclude that the concentrations of trace elements found do not pose a toxicological risk in the studied intake scenarios. However, in some cases, such as Li, Al, or Pb, it could be interesting to consider the overall dietary intake, as they could be an important part of the contribution to toxicity.

## 4. Conclusions

Dried fruits, as well as their source foods (fruits), can be a source of different metals and semimetals which may be present in trace amounts, especially derived from the way they are processed due to the concentration of their components. The concentration of Al and B stands out, especially in sultanas and plums. However, the highest contribution percentages to the reference value are made by Li in all cases, with contribution percentages of 18.6–35.3%.

The dried fruit with the highest overall concentrations of the elements analysed was figs, except for B (plums), Pb (plums), Li (plums), V (sultanas), and Al (sultanas). The dried fruit with the lowest concentrations was dates, except for Al (apricot kernels).

Thus, the results obtained in this study allow us to say that the dried fruits marketed in Spain do not present concentrations of the elements analysed (Al, B, Ba, Cd, Cr, Li, Ni, Pb, Sr, and V) that could pose a toxicological risk in the amounts recommended for their consumption (30 g/day) in order to obtain the beneficial effects attributed to them. Caution is advised for dried fruits from other regions with higher concentrations of these elements until a proper risk assessment of their consumption has been carried out.

## Figures and Tables

**Table 1:** Types of dried fruit samples selected for the study.

Type	No. Samples	Country Origin	Sample Collection Point
Plums	9	Spain	Tenerife, Canary Islands (Spain)
Dates	8	Tunisia	
Sultanas	9	Turkey	
Apricot kernels	8	Spain	
Figs	8	Spain	
Total	42		

**Table 2:** Limits of detection (LOD), limits of quantification (LOQ) and instrumental wavelength of the ICP used for the study.

Element	LOD (mg/L)	LOQ (mg/L)	Wavelength (nm)
B	0.008	0.027	249.6
Ba	0.0006	0.002	455.4
Cr	0.001	0.005	267.7
Sr	0.003	0.011	407.7
Li	0.013	0.031	670.8
Ni	0.0009	0.003	221.6
V	0.0014	0.004	292.4
Al	0.005	0.015	167.0
Cd	0.0007	0.002	214.4
Pb	0.0009	0.003	220.3

**Table 3:** Adult intake limit values for the elements analysed, as set by the main health organisations.

Element	Parameter	Intake Limit Value	Organization	Reference
Cd	TWI	2.5 μg/kg bw/week	EFSA	[20]
Pb	BMDL	0.50 μg/kg bw/day ^1^ 0.63 μg/kg bw/day ^2^ 1.50 μg/kg bw/day ^3^	EFSA	[21]
Al	TWI	1 mg/kg bw/week	EFSA	[33]
Cr	TDI	0.3 mg/kg bw/day	EFSA	[23]
Li	p-RfD	2 μg/kg bw/day	EFSA	[26]
Ni	TDI	13 μg/kg bw/day	EFSA	[24]
Sr	TDI	0.13 mg/kg bw/day	WHO	[25]
B	UL	10 mg/day	EFSA	[34]
V	RfD	7 μg/kg bw/day	EFSA	[26]
Ba	TDI	0.2 mg/kg bw/day	EFSA	[26]

^1^ For neurotoxicity; ^2^ for nephrotoxicity; ^3^ for cardiovascular effects.

**Table 4:** Mean (Conc.) concentrations (mg/kg fresh weight), standard deviations (SD), and maxima and minima of the analysed samples.

Element	Prunes		Dates		Sultanas		Apricot Kernels		Figs	
	Conc. ± SD	Min–Max	Conc. ± SD	Min–Max	Conc. ± SD	Min–Max	Conc. ± SD	Min–Max	Conc. ± SD	Min–Max
Al	9.94 ± 3.94	1.74–16.3	7.23 ± 4.36	2.26–14.5	12.7 ± 5.13	5.14–12.2	6.12 ± 2.66	3.55–11.9	8.22 ± 3.75	3.21–15.1
B	6.26 ± 4.45	1.07–14.4	2.72 ± 0.78	1.18–4.07	5.59 ± 2.85	1.45–12.2	3.81 ± 2.22	0.52–8.11	5.24 ± 1.23	3.37–7.62
Ba	1.45 ± 1.91	0.22–8.50	0.50 ± 0.32	0.12–1.23	0.74 ± 0.26	0.36–1.41	0.94 ± 0.47	0.57–2.31	2.32 ± 0.90	0.43–4.11
Cd	0.003 ± 0.0005	<LOQ–0.01	0.003 ± 0.0009	<LOQ–0.01	0.003 ± 0.0008	<LOQ–0.01	0.003 ± 0.001	<LOQ–0.01	0.02 ± 0.01	<LOQ–0.05
Cr	0.10 ± 0.13	0.04–0.65	0.04 ± 0.02	0.02–0.08	0.07 ± 0.03	0.04–0.12	0.05 ± 0.03	0.02–0.13	0.11 ± 0.06	0.04–0.20
Li	1.61 ± 1.22	<LOQ–4.15	0.85 ± 0.68	<LOQ–2.28	1.45 ± 1.08	<LOQ–4.08	1.01 ± 0.95	<LOQ–2.77	1.26 ± 1.03	<LOQ–3.86
Ni	0.15 ± 0.10	0.05–0.43	0.07 ± 0.02	0.04–0.10	0.07 ± 0.02	0.04–0.12	0.39 ± 0.22	0.04–0.79	0.75 ± 0.41	0.25–1.39
Pb	0.04 ± 0.03	0.01–0.10	0.02 ± 0.02	0.01–0.07	0.03 ± 0.01	0.01–0.05	0.03 ± 0.01	0.01–0.06	0.03 ± 0.01	0.02–0.05
Sr	2.25 ± 1.52	<LOQ–6.00	1.97 ± 1.37	<LOQ–4.59	2.80 ± 1.58	1.20–6.10	2.10 ± 1.44	<LOQ–4.83	2.79 ± 2.14	<LOQ–5.14
V	0.01 ± 0.002	0.01–0.02	0.01 ± 0.0003	0.01–0.01	0.03 ± 0.03	0.01–0.11	0.03 ± 0.05	0.01–0.17	0.01 ± 0.0003	0.01–0.01

**Table 5:** Comparison of trace elements in dried fruits from different countries (mg/kg).

Reference (Origen)	Dried Fruit	Toxic or Trace Elements			
		Al	Cd	Ni	Pb
Duran et al., 2008 (Turkey) [35]	Apricot	5.78	0.81	-	-
	Figs	4.31	0.28	-	-
	Plum	3.58	0.45	-	-
	Plum	9.40	0.63	-	-
	Black grapes	2.12	0.63	-	-
Altundag and Tuzen, 2011 (Turkey) [36]	Apricot	1.22	0.16	1.51	1.45
	Figs	0.83	0.32	2.26	0.40
	Yellow plum	1.03	0.23	0.95	0.56
	Black plum	2.71	0.12	0.74	0.41
	Raisins	7.69	0.21	1.41	0.67
Mehta et al., 2014 (India) [39]	Plum	-	-	1.08	0.21
Ivanovic et al., 2016 (Serbia) [40]	Apricot	0.77	-	0.40	0.72
	Dates	0.36	-	0.08	0.71
	Figs	0.32	-	1.07	0.41
	Plum	0.72	-	0.72	0.92
Al-Massaedh et al., 2018 (Jordan) [18]	Black raisins	-	0.03	0.21	0.37
	Figs	-	0.02	0.29	0.29
	Apricot	-	0.05	0.26	0.57
	Plum	-	0.02	0.21	0.25
Kulluk et al., 2023. (Turkey) [38]	Plum	-	0.08	2.75	1.12
	Dates	-	0.19	2.80	2.02
	Black raisins	-	0.12	3.31	2.01
	Apricot	-	0.13	2.92	1.68
	Figs	-	0.15	3.26	2.50

**Table 6:** Risk assessment of trace element intake from the recommended consumption of each of the dried fruits included in the study.

Sample	TDI (mg/kg bw/Day)								UL (mg/Day)		RfD (mg/Day)		BMDL (µg/kg bw/Day)			Prf-D (µg/kg bw/Day)		TWI (mg/kg bw/Week)			
	Ba		Cr		Ni		Sr		B		V		Pb			Li		Al		Cd	
	EDI (mg/Day)	%	EDI (µg/Day)	%	EDI (mg/Day)	%	EDI (mg/Day)	%	EDI (mg/Day)	%	EDI (mg/Day)	%	EDI (µg/Day)	% *	% **	EDI (µg/Day)	%	EWI (mg/Week)	%	EWI (µg/Week)	%
Plums	0.44	0.32	3.00	0.01	4.50	0.51	0.07	0.76	0.19	0.94	0.0003	0.06	1.20	2.78	1.17	48.3	35.3	2.09	3.05	0.63	0.37
Dates	0.02	0.11	1.20	0.01	2.10	0.24	0.06	0.66	0.08	0.41	0.0003	0.06	0.60	1.39	0.58	25.5	18.6	1.52	2.22	0.63	0.37
Sultanas	0.02	0.16	2.10	0.01	2.10	0.24	0.08	0.94	0.17	0.84	0.001	0.21	0.90	2.09	0.88	43.5	31.8	2.67	3.89	0.63	0.37
Apricot kernels	0.03	0.21	1.50	0.01	11.7	1.31	0.06	0.71	0.11	0.57	0.001	0.21	0.90	2.09	0.88	30.3	22.1	1.29	1.88	0.63	0.37
Figs	0.07	0.51	3.30	0.02	22.5	2.53	0.08	0.94	0.16	0.79	0.0003	0.06	0.90	2.09	0.88	37.8	27.6	1.73	2.52	4.20	2.45

* For the BMDL of Pb of 0.63 μg/kg bw/day; ** for Pb BMDL of 1.50 μg/kg bw/day.

## Data Availability

The original contributions presented in the study are included in the article, further inquiries can be directed to the corresponding author.
